# Subjective Health and Personal Values in Immigrants and Nonimmigrants Across Europe: Evidence From the COVID-19 Era

**DOI:** 10.5964/ejop.17957

**Published:** 2026-02-27

**Authors:** Hwoyeon Seo, Eunlak Kim, Hong Min Kim, Joon Hyung Jung, Sanghoon Oh, Jae-Suk Yang, Jiho Cha

**Affiliations:** 1 Graduate School of Future Strategy, Korea Advanced Institute of Science and Technology (KAIST), Daejeon, Republic of Korea; 2 Center for Technology and Behavioral Health, Geisel School of Medicine, Dartmouth College, Lebanon, NH, USA; 3 Korea Institute of Oriental Medicine (KIOM), Daejeon, Republic of Korea; 4 SNU College of Medicine, Major in History of Medicine & Medical Humanities, Seoul, Republic of Korea; 5 Department of Psychiatry, College of Medicine, Chungbuk National University, Republic of Korea; 6 Department of Psychiatry, Uijeongbu Eulji Medical Center, Eulji University School of Medicine, Republic of Korea; 7 National Assembly of the Republic of Korea, Seoul, Republic of Korea; University of Western Australia, Perth, Australia

**Keywords:** immigrant, subjective health, personal values, European Social Study, pandemic

## Abstract

COVID-19 has profoundly impacted physical and mental health worldwide, disproportionately affecting vulnerable populations, including immigrants. While subjective health (SH) is widely used as a measure of well-being, little is known about how personal values influence SH differently between immigrants and nonimmigrants during crises. This study explores the relationship between personal values and SH, focusing on differences between immigrant and nonimmigrant groups. Using data from the European Social Survey (ESS), we analyzed responses from 32,963 individuals across 20 countries (Round 10: 2020–22). Multilevel modeling showed that Axis_open (*Openness to change* over *Conservation*) was positively associated with SH in both immigrant and nonimmigrant groups. However, Axis_self (*Self-transcendence* over *Self-enhancement*) was not significant among nonimmigrants, while in the immigrant group, higher Axis_self scores were significantly associated with poorer SH. These findings underscore the importance of considering cultural and migratory contexts when addressing the health implications of personal values.

COVID-19 pandemic had profound impacts on every aspect of life of human beings globally including physical and mental health. The pandemic was reported to have been associated with 14.9 million excess deaths in 2020 and 2021 (World Health Organization, 2022), and a number of studies highlighted more people experienced fear, emotional disturbance, sleep problems, anxiety and depression especially during the early stage of the pandemic (Marques de Miranda et al., 2020; Usher et al., 2020). However, the effect of the pandemic on health was not equally distributed across all populations (Bambra et al., 2020). Vulnerable groups with low socioeconomic resources at both the individual and national levels were identified as being the most threatened by the pandemic (Abedi et al., 2021; Mishra et al., 2021). Often, these disadvantages — economic deprivation, limited access to health care, and ethnic minority status — are interconnected and overlapping (Diener et al., 2017). In line with ethnic disparities, immigrants tend to be overrepresented in frontline jobs. For example, foreign-born workers make up 19% of U.S workers in frontline industries like health care, social services, grocery stores, and agriculture, underscoring the need for tailored attention to those groups (Gelatt, 2020).

Subjective health (SH), or individual’s self-assessment of their overall health, is a widely used measure in epidemiological and psychosocial research. SH reflects an integration of objective health information, physical sensations, and subjective perceptions, encompassing psychological and evaluative components (Liang, 1986). Due to its validity and cost-effectiveness, SH serves as a valuable health indicator, both on its own and in combination with clinical data (Ferraro et al., 1997; Ware et al., 1978). Beyond its practical advantages, studies have shown that people with poor SH are more likely to die prematurely, even if they have no apparent medical problems. They are also more likely to suffer from chronic diseases, such as heart disease, stroke, and cancer (Benjamins et al., 2004). Since the COVID-19 pandemic emerged as a major public health challenge, the urgent need to understand physical and mental health in disaster situations has been emphasized. SH, which represents an individual's mental understanding of their physical health, has re-emerged as an important concept to bridge the gap between these dimensions and understand their interaction.

Since health exists within a complex and interconnected system affected by genetic, psychological, physical and social environments, there has been growing interest on relationship between health and personal values. For example, Aavik et al. (2014) showed that the utilization of health services is related to personal values. Individuals’ values regarding health interact with other personal values to shape health-related attitudes, intentions and behaviors influencing perceived health outcomes (Aavik & Dobewall, 2017; Young & West, 2010).

Schwartz’s Theory of Personal Value posits that values are broad, guiding principles that influence human behavior and decision-making across diverse contexts (Schwartz, 2012). Schwartz’s refined model organizes basic human values along two bipolar dimensions — self-enhancement vs self-transcendence and openness to change vs conservation — which have been empirically validated across cultures. Personal values include categories such as benevolence, achievement, tradition, and hedonism, reflecting distinct motivational goals. Importantly, Schwartz argues that values are hierarchically organized, with individuals prioritizing certain values over others depending on their cultural, social, and personal contexts. Several studies have investigated the relationship between SH and specific personal values, such as hedonism (Williams et al., 2018) or benevolence Åberg et al. (2026). For example, Åberg et al. (2026) reported a positive relationship between compliance with preventive measures and individuals’ degree of benevolence during the COVID-19 Pandemic in Sweden. However, limited attention has been given to how personal values might influence SH differently between immigrants and nonimmigrants, in particular during the COVID-19 pandemic. To address this gap, this exploratory study aims to investigate the differential effects of personal values on SH between immigrants and nonimmigrants using data from the European Social Study (ESS). By focusing on values identified in Schwartz’s Theory and their interaction with immigrant status, this research seeks to illuminate pathways through which values may influence health disparities exacerbated by the pandemic. Such insights are crucial for informing targeted interventions that mitigate the unequal health impacts experienced by vulnerable populations.

## Method

### Dataset

In this study, the European Social Survey (ESS) data were used. The ESS is a cross-national survey that has been conducted every two years since its establishment in 2001. The ESS focuses on the social, political, moral, and psychological structure of Europe. The questions in the ESS are carefully designed to gather information about respondents’ social behavior and attitudes.

The ESS Round 10 covers 31 countries, including 22 countries where data were collected through face-to-face interviews and 9 countries that employed web-based self-completion methods due to the COVID-19 pandemic. To minimize potential bias stemming from different interview methods, we included only the countries where face-to-face interviews were conducted. Furthermore, France and Montenegro did not provide information on the history of COVID-19 infection, which resulted in exclusion from the analysis. As a result, the final analysis included 20 countries with 34,356 individuals.

### Measure

#### Outcome: Subjective Health

In the ESS, there is a single question asking respondents to subjectively assess their general health on a 5-point Likert scale, ranging from 1 (“Very good”) to 5 (“Very bad”). In this study, this item was reverse-coded and used as the outcome measure.

#### Predictors: Personal Values

In the ESS, 21 survey questions were designed to measure respondents’ personal values based on Schwartz’s Theory of Basic Values (Lindeman & Verkasalo, 2005; Schwartz, 2012). The theory provides a robust framework for understanding how values shape actions, attitudes, and well-being across diverse populations, including immigrants and non-immigrants. Respondents answered each question using a 6-point Likert scale, where 1 indicated “Very much like me” and 6 indicated “Not at all like me”. However, to ensure ease of interpretation, the 21 items were reverse-coded so that 1 indicates “Not at all like me” and 6 indicates “Very much like me” before analysis.

Since hedonism spans both ‘Self-enhancement’ and ‘Openness to change’ in the original theory, Cronbach’s alphas were used to determine the category to which Hedonism was most strongly associated in this sample. When the two items measuring hedonism (‘important to have a good time’ and ‘important to seek fun and things that give pleasure’) were excluded from both categories, the alpha values dropped from 0.73 to 0.72 in Self-enhancement, and from 0.76 to 0.71 in Openness to change. Based on these findings, Hedonism was categorized under Openness to change.

To adjust for individual-level tendencies, all 21 value items were ipsatized by subtracting each respondent’s mean score across the items (Bartram, 1996). For parsimony and theoretical clarity in the final model, two orthogonal value axes were constructed to reflect fundamental motivational tensions in Schwartz’s theory (Schwartz, 2012). Axis_self, calculated as the difference between self-transcendence and self-enhancement, captures the degree to which individuals prioritize altruistic values over self-focused goals. Axis_open, derived by subtracting conservation from openness to change, reflects a preference for autonomy and innovation over stability and tradition.

#### Control Variables: Immigration, COVID-19 Experience and Demographic Information

The ESS does not directly ask about immigration status, but there are questions that allow immigration status to be inferred. For example, the survey has questions as to whether the respondent was born in the country where they currently live, whether they are a citizen of the current country and how long they have lived in the current country since their first arrival.

For this study, it would be inappropriate to define all participants who were not born in the current country as immigrants, as many previous studies have pointed out the importance of age at immigration on various outcomes (Beck et al., 2012; Corak, 2012; Schaafsma & Sweetman, 2001). While this remains open to ongoing debate, since the development of one’s value exists on a continuum, some studies have indicated that people who migrate after the age of 6 have distinct outcomes compared to natives, such as differences in dropout rates and educational attainment (Cohen Goldner & Epstein, 2014; Gonzalez, 2003). In the light of evidence, we define immigrants as people who were not born in the current country and moved there after the age of 6.

Individual-level demographic information that is available from the ESS includes respondents’ age, gender, education level, and current cohabitation status with a partner. Education level was categorized using UNESCO’s ISCED from 0 (No primary education) to 800 (Doctoral degree) and simplified to 9 levels by their first digit (0–8). Cohabitation status was categorized based on the respondent’s answer to the question regarding relationship with a spouse or a partner who is currently living with them. Participants who answered yes were classified as ‘Living together’; others were classified as ‘Living alone’. The variable measuring respondents’ total net household income was ranked on a scale from 1 to 10 by decile.

In addition, we assumed that the COVID-19 experience would have a significant impact on the SH, so a question inquiring whether the respondent had experienced COVID-19 was included as an independent variable. Respondents who answered ‘Yes’ to either having had coronavirus themselves or having family members who had coronavirus were classified as exposed to COVID-19.

#### Statistical Analysis

The analysis was conducted using the lme4 package (Version 1.1.35.5) in the R statistical software (Version 4.4.1). Before the data analysis, the dataset was prepared and cleaned for analysis. In the case of income decile, 7,888 missing values (22.9%) were identified. Simply omitting these values could have significantly biased the results, so we used predictive mean matching (PMM) algorithm of the multivariate imputation by chained equations (MICE) method to handle the missing data. The distributions of income deciles before and after imputation were compared and found not to be statistically significantly different. All other missing values were excluded from the analysis (4% of cases).

To explore the differences between immigrants and non-immigrants in key variables such as education, age, sex, household income, cohabitation status, personal values, COVID-19 experience and SH, we performed generalized linear mixed models for binary outcomes, cumulative link mixed models for ordinal outcomes, and linear mixed models for continuous outcomes. These tests assessed whether significant associations existed between immigration status and the selected variables. Next, given the nested structure of the data, with individuals grouped by country, we used multi-level mixed-effects models to analyze the relationship between personal values and SH.

The modeling process consisted of three steps. We first fitted a null model (M0) that included only random intercepts for countries. This model was used to assess the proportion of variance in SH attributable to differences between countries. In Model 1 (M1), we included two orthogonal value dimensions (Axis_self and Axis_open) as fixed-effect predictors, along with immigration status and their interaction terms, to test whether the associations between personal values and SH differed for immigrants versus immigrants. We also included exposure to COVID-19, given its well-documented impact on health during the pandemic. Finally, we fitted an adjusted model (M2) that included all variables from the previous step and all additional potential confounding variables, including education, age, sex, household income, cohabitation status and national GDP.

All continuous variables (the dependent variable, SH; the two axes; age; income; education; and GDP) were standardized to z-scores (*M* = 0, *SD* = 1), while binary predictors (immigration status, gender, cohabitation status, COVID-19 exposure) remained in their original 0/1 coding so that their coefficients represent the effect of the full-category contrast.

All models accounted for the multi-level structure of the data by including random intercepts for countries. Model parameters were estimated using restricted maximum likelihood (REML). Statistical significance was assessed at a p-value threshold of 0.05.

All data, code, and materials for this study are available at Seo (2026).

## Result

### Descriptives

The analysis was conducted with the final sample of 32,963 after removing missing values. Table 1 presents a comparison between immigrants (*N* = 2,108) and non-immigrants (*N* = 30,855) across demographic, personal values, and health-related variables. No significant gender differences were observed (*p* = .461) but age distribution differed significantly (*p* < .001), with immigrants having a higher proportion of individuals aged 20–39 and fewer aged 60+. Immigrants were less likely to have lower education levels (66.8% vs 73.0%) and had higher rates of advanced degrees (21.2% vs 13.3%, *p* < .001). Households with income above the 5^th^ decile were more common among non-immigrants (46.8% vs 42.8%, *p* < .001). Immigrants were also less likely to live with a partner (*p* < .001). For personal values, immigrants scored significantly higher on all values. The reported COVID-19 exposure was not significant (34.7% vs 29.5%, *p* = .056). Regarding SH, there was no difference between two groups (*p* = .357).

**Table 1:** Descriptive Variables of ESS by Immigrant and Non-immigrant Group

	Immigrant	Non-immigrant	
Variable	(*N* = 2,108)	(*N* = 30,855)	*p*-value
Gender (*n*, %)			.461
Male	978 (46.4%)	14,184 (45.9%)	
Female	1,130 (53.6%)	16,671 (54.1%)	
Age group (*n*, %)			<.001
<20	54 (2.6%)	1,323 (4.3%)	
20–39	610 (28.9%)	7,754 (25.1%)	
40–59	797 (37.8%)	10,518 (34.1%)	
60+	647 (30.7%)	11,260 (36.5%)	
Education level (*n*, %)			<.001
Post-secondary/Tertiary or less	1,409 (66.8%)	22,524 (73.0%)	
Bachelor’s or equivalent	253 (12.0%)	4,221 (13.7%)	
Master’s or equivalent	392 (18.6%)	3,796 (12.3%)	
Doctoral or equivalent	54 (2.6%)	314 (1.0%)	
Households’ total income (*n*, %)			<.001
Less than 5^th^ decile	1,206 (57.2%)	16,406 (53.2%)	
Greater than 5^th^ decile	902 (42.8%)	14,449 (46.8%)	
Living with partner(s) (*n*, %)			<.001
Living with partner(s)	777 (36.9%)	13,472 (43.7%)	
Living alone	1,331 (63.1%)	17,383 (56.3%)	
Personal Values (*M*, *SD*)			
Self Transcendence	4.96 (0.70)	4.79 (0.75)	<.001
Openness	4.19 (0.84)	4.07 (0.89)	.018
Conservation	4.41(0.80)	4.32 (0.81)	.012
Self Enhancement	3.67 (0.98)	3.65 (1.01)	.002
COVID-19 Experience (*n*, %)			.056
Yes	731 (34.7%)	9,105 (29.5%)	
No	1,377 (65.3%)	21,750 (70.5%)	
Subjective Health (*n*, %)			.357
Good	1,516 (71.9%)	20,988 (68.0%)	
Fair	459 (21.8%)	7,599 (24.6%)	
Bad	133 (6.3%)	2,268 (7.4%)	

*Note*. *p*-values are derived from generalized linear mixed models for binary outcomes, cumulative link mixed models for ordinal outcomes, and linear mixed models for continuous outcomes.

### Main Results

Table 2 presents the results of multilevel models predicting SH, based on weighted data. The Intraclass Correlation Coefficient (ICC), calculated as the proportion of total variance explained by country-level differences, was 6.87%, indicating that country-level clustering accounted for a small but meaningful proportion of the variance in SH. The null model (M0) includes only the intercept, estimating the mean SH score as 3.90 (*p* < .001). The unadjusted model (M1) showed that Axis_self was negatively associated with the SH (*b* = -0.03, *p* < .001), while Axis_open had a positive relationship (*b* = 0.19, *p* < .001). However, among immigrants, the positive association between Axis_open and SH was weaker, as indicated by a significant interaction effect (*b* = -0.03, *p* = .038). This suggests that the strong positive relationship observed in non-immigrants was reduced for immigrants.

**Table 2:** Multilevel Model Results: Predicting Subjective Health (Weighted)

	Null Model, M0	Unadjusted Model, M1	Adjusted Model, M2	Standardized β of M2 model
Variable	(b (*SE*))	(b (*SE*))	(b (*SE*))	(β (*SE*))
Fixed Effects				
Intercept	3.90 (0.04)***	3.96 (0.04)***	4.22(0.06)***	-0.01 (0.05)
Personal Values				
Axis_self		-0.03 (0.01)***	0.00 (0.01)^a^	0.00 (0.01)^a^
Axis_open		0.19 (0.01)***	0.08 (0.00)***	0.09 (0.01)***
Immigration status		0.20 (0.03)***	0.11 (0.02)***	-0.01 (0.02)
Exposure to Covid19		0.07 (0.01)***	-0.07 (0.01)***	-0.07 (0.01)***
Interaction terms (with immigration status)				
X Axis_self		-0.10 (0.02)***	-0.10 (0.01)***	-0.13 (0.02)***
X Axis_open		-0.03 (0.02)*	0.01 (0.02)	0.01 (0.02)
Covariates				
Age (year)			-0.02 (0.00)***	-0.3 (0.01)***
Gender (female)			-0.05 (0.01)***	-0.05 (0.01)***
Income (decile)			0.04 (0.00)***	0.12 (0.01)***
Education			0.05 (0.00)***	0.10 (0.01)***
GDP (Million Euros)			0.02 (0.05)	0.01 (0.04)
Living with partners			0.07 (0.01)***	0.07 (0.01)***

*Note.* ^a^Coefficients or standard errors smaller than 0.005 are rounded to 0.00.

*p < .1. **p < .01. ***p < .001.

**Figure 1:**
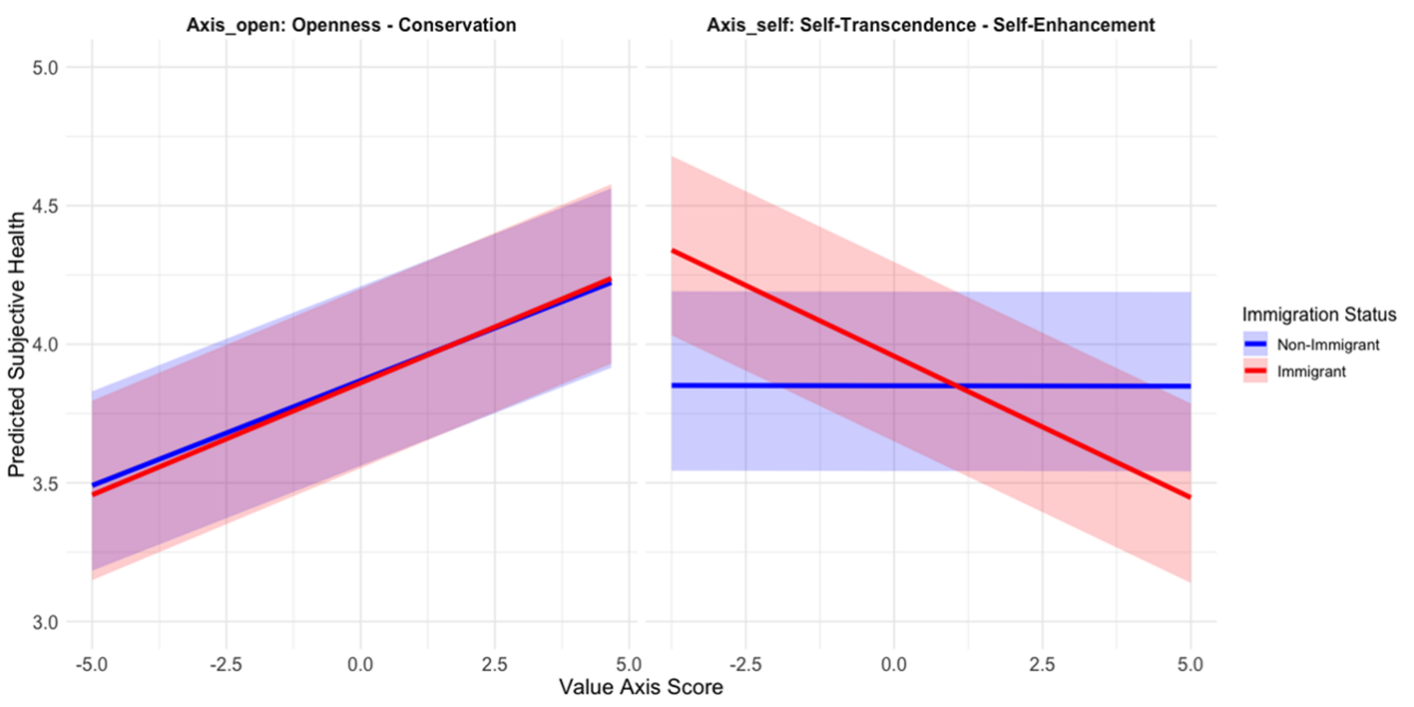
Interaction Plots Between Personal Values and Subjective Health in the Adjusted Model (M2) *Note.* The shade means 95% of variation of random intercepts by countries.

In the adjusted model (M2), the effects of Axis_open was attenuated but remained significant (β = 0.09, *p* < .001) while Axis_self was no longer significant among nonimmigrants. Interaction terms revealed that the impact of personal values on SH differed by immigration status. Specifically, the effect of Axis_self became significantly negative only among immigrants (β = -0.13, *p* < .001), as indicated by the interaction term. Similarly, the positive effect of Axis_open was weaker among immigrants due to negative interaction term (see Figure 1).

Other covariates also showed the significant association with SH. Older respondents reported poorer SH (β = -0.3, *p* < .001), and women were more likely than men to report poorer health (β = - 0.05, *p* < .001). By contrast, higher income (β = 0.12, *p* < .001), higher education (β = 0.10, *p* < .001) and living with a partner (β = 0.07, *p* < .001), were each associated with better SH. National GDP, however, was not significantly associated with SH.

### Model Evaluation

Table 3 presents the performance metrics for the null M0, unadjusted M1, (i.e., including personal values) and adjusted M2 models (i.e., including personal values and covariates), including AIC, BIC and conditional *R*^2^, which represents the explained variance attributed to fixed and random effects. The adjusted model (M2) showed the best fit, with an AIC of 101,425.5, BIC of 101,551.6 and a conditional *R*^2^ of 0.363, indicating 36.3% of the variance in SH was explained by the model. In comparison, the null M0 model (AIC = 109,306.7, BIC = 109,231.9, *R*^2^ = 0.069) and the unadjusted M1 model (AIC = 106,965.2, BIC = 107,040.8, *R*^2^ = 0.171) had poorer fit.

**Table 3:** model performance by AIC, BIC and R^2^

Model	AIC	BIC	*R*^2^ (Conditional*)*	*SD* (Intercept: country)	*SD* (Residual)
Null Model (M0)	109,206.7	109,231.9	0.07	0.18	0.68
Unadjusted Model (M1)	106,965.2	107,040.8	0.17	0.18	0.66
Adjusted Model (M2)	101,425.5	101,551.6	0.36	0.19	0.60

*Note.* AIC = Akike’s information criterion; BIC = Bayesian information criterion; Conditional *R*^2^ indicates the proportion of variance explained by both fixed and random effects.

To assess multicollinearity in the adjusted model, two tests were performed. First, a correlation matrix of the independent variables showed all pairwise correlations were below 0.5, indicating that multicollinearity was minimal. Second, the Generalized Variance Inflation Factor (GVIF) values for all fixed effects were calculated, with adjusted GVIF values for all predictors below 5, confirming that multicollinearity was not a major concern.

Residual diagnostics were conducted to evaluate model assumptions. The Q-Q plot of the residuals showed slightly heavy tails compared to a normal distribution. However, this minor deviation did not compromise the validity of the model, as the assumption of homoscedasticity was upheld. Furthermore, a box plot of residuals by country revealed that the residuals were centered around zero for most countries, indicating unbiased predictions. The spread of residuals across countries was consistent, further supporting the assumption of homoscedasticity. Overall, the adjusted model demonstrated a good fit to the data, effectively explaining variance in SH while satisfying key model assumptions.

## Discussion

Following Schwartz’s well-known circumplex, we collapsed the ten basic values into two bipolar axes: Axis_self (Self-transcendence over Self-enhancement) and Axis_open (Openness to Change over Conservation). These axes capture the core motivational trade-offs that guide people’s priorities. The key finding of this study is that Subjective Health (SH) showed positive associations with personal values of Axis_open in both immigrant and non-immigrant groups, while Axis_self was negatively associated with SH only among immigrants.

Although previous literature on the role of personal values in SH is relatively scarce, some studies highlighted the relationship between personal values and health-related behaviors or subjective well-being (SWB), which are closely related to SH (Ngamaba et al., 2017). Although Schwartz postulated that Openness, which conveys person-focused growth needs and the value of Self-transcendence would be positively related to SWB, many studies reported inconsistent findings regarding the relationship between SWB and personal values (Buchanan & Bardi, 2015; Sortheix & Schwartz, 2017). In contrast, the negative association between Power and SWB has been reported consistently, aligning with the relationships observed between health-related behaviors and personal values.

For example, a study conducted in Australia showed Universalism was positively associated with healthy eating habits, while Hedonism was related to overeating (Wang et al., 2012). Another study reported that risky sexual behaviors were associated with values such as Achievement, Power, Hedonism, Stimulation and Self-direction (Goodwin et al., 2002). Consistent with these findings, a study involving more than 100,000 Finnish citizens suggested that the value for Power showed significant negative associations with unhealthy behaviors, while Universalism was linked to regular exercise and healthy eating habit (Honka et al., 2019).

The negative relationship between Axis_self and SH in the immigrant group may appear inconsistent with previous findings. This could be understood through the lens of self-enhancement and self-protection mechanisms. Immigrants often face unique stressors, such as social exclusion, economic instability, and cultural displacement, which can be exacerbated during crises (Solheim et al., 2022). In such contexts, values related to Self-enhancement — such as striving for power and achievement — might function as effective coping strategies (Dufner et al., 2019). These strategies may help maintain a sense of agency and psychological stability, although their effectiveness and long-term impact on SH may vary depending on individual circumstances.

Cultural dynamics would also play a role. Immigrants often navigate a dual framework, balancing their cultural values with those of the dominant society. In individualistic cultures that prioritize personal achievement and power, adopting Self-enhancement values could facilitate social integration and acceptance. Such alignment may not only help immigrants adapt socially but also support their psychological well-being by reducing feelings of cultural dissonance (Alicke & Sedikides, 2009).

The use of personal values as a coping mechanism could be supported by a study investigating the changes in personal values in Australia during the COVID-19 pandemic. Daniel et al. reported that Openness to change and Self-enhancement decreased but reversed at the height of the pandemic. Conversely, Self-transcendence values decreased, and this change was prominent among groups concerned about the pandemic (Daniel et al., 2022).

The findings of this research may offer tentative insights into supporting immigrant health and psychological well-being, particularly during crises such as the COVID-19 pandemic. Although the association between Axis-self and SH among immigrants was statistically significant, the effect size was relatively small, suggesting that its practical impact may be limited. Nevertheless, this association may reflect subtle ways in which values like self-empowerment and personal agency relate to psychological adaptation. Interventions that gently encourage goal-setting, skills development, and recognition of individual efforts could still be beneficial in fostering a sense of control and competence. A recent review on interventions for empowering immigrant women highlighted that psychoeducation and cognitive restructuring strategies showed the most promising results (Silva & Pereira, 2023).

Some limitations of this study should be noted. First, while this survey was conducted face-to-face to enhance its reliability, countries using the web-based survey were excluded from the analysis. It is possible that these countries were more severely impacted by the pandemic, which could introduce systematic bias into the results. Second, our cross-sectional design not only limits the ability to draw causal inferences but also underscores the need for future longitudinal research to track how personal values and SH evolve over time. Third, the immigrant population analyzed in this study is heterogeneous and encompasses diverse subgroups with distinct experiences. Additionally, existing evidence suggests gender differences in coping with crises (Bojanowska et al., 2021). This diversity could impact on the tendency for responses. However, to maintain the focus on the differences between immigrants and non-immigrants, gender-based analyses were not conducted, representing an opportunity for future research. Fourth, while this study hypothesized linear relationships between variables, it is possible that more complex, non-linear relationships exist, which warrants further exploration. Finally, because all interviews were face-to-face, respondents’ value orientations may have influenced how they reported their health (for example, highly self-enhancing individuals might under-report poor health), introducing potential response biases tied to the survey’s micro-setting.

### Conclusion

This study highlights the complex relationship between value systems and SH among immigrant populations in Europe during the COVID-19 pandemic. Specifically, greater endorsement of Axis_self (reflecting Self-transcendence over Self-enhancement) was associated with lower SH among immigrants, while Axis_open (Openness to Change over Conservation) was positively associated with SH across both immigrant and nonimmigrant groups. Although these associations were statistically significant, the effect sizes were modest, suggesting that personal values may play a subtle yet meaningful role in psychological well-being during times of crisis. These findings underscore the importance of culturally informed public health strategies that consider value orientations when supporting immigrant resilience and adaptation under global stressors.

## Supplementary Materials

**Table:** 

Type of supplementary materials	Availability/Access
Data	
Final dataset.	Seo (2026)
GDP Europe dataset.	Seo (2026)
ESS10.	Seo (2026)
Code	
ESS EJOP R code.	Seo (2026)
Material	
ESS10 source questionnaires.	Seo (2026)
Study/Analysis preregistration	
The study was not preregistered.	—
Other	
ESS10 codebook.	Seo (2026)
Codebook summary.	Seo (2026)
Readme file.	Seo (2026)
Revision 1.	Seo (2026)

## Data Availability

The data, code, and materials for this study are available at Seo (2026).
